# Genetic Spectrum of Lithuanian Familial Hypercholesterolemia Patients

**DOI:** 10.3390/jcdd12050197

**Published:** 2025-05-21

**Authors:** Urte Aliosaitiene, Rimante Cerkauskiene, Aleksandras Laucevicius, Migle Vilniskyte, Viktoras Sutkus, Antanas Mainelis, Birute Burnyte, Jurate Barysiene, Zaneta Petrulioniene

**Affiliations:** 1 Institute of Clinical Medicine, Faculty of Medicine, Vilnius University, 08661 Vilnius, Lithuania; rimante.cerkauskiene@santa.lt (R.C.); migle.vilniskyte@gmail.com (M.V.); visutkus@gmail.com (V.S.); jurate.barysiene@santa.lt (J.B.); zaneta.petrulioniene@santa.lt (Z.P.); 2 State Research Institute, Centre for Innovative Medicine, 01513 Vilnius, Lithuania; aleksandras.laucevicius@santa.lt; 3 Faculty of Mathematics and Informatics, Vilnius University, 08412 Vilnius, Lithuania; amgpastas@gmail.com; 4 Institute of Biomedical Sciences, Faculty of Medicine, Vilnius University, 03101 Vilnius, Lithuania; birute.burnyte@santa.lt

**Keywords:** familial hypercholesterolemia, genetic variants, variant of uncertain significance, atherosclerotic cardiovascular disease

## Abstract

Background and aims: Although familial hypercholesterolemia (FH) is a common congenital cause of elevated low-density lipoprotein cholesterol (LDL-C), it remains underdiagnosed and undertreated worldwide due to its inherent genetic heterogeneity. This study aimed to determine the prevalence of genetic variants in a Lithuanian patient cohort with clinically diagnosed FH and evaluate their possible clinical implications. Methods: A total of 172 patients were included in the retrospective analysis. The study population comprised males and females ranging from 0 to 85 years of age, with LDL-C levels exceeding 4.9 mmol/L in adults and 3.9 mmol/L in children. The subjects were divided into four groups according to the Dutch Lipid Clinic Network (DLCN) criteria (definite, probable, possible, and unlikely). Children were analyzed separately. Next-generation sequencing (NGS) has been chosen as the most appropriate technique for genetic testing. All identified variants were categorized into three groups: (1) pathogenic, (2) likely pathogenic, and (3) variants of uncertain significance. Subjects without detected variants were classified into group (4) No mutation. Results: Women were diagnosed with FH significantly later than men (*p* = 0.033). Genetic testing identified FH-causing variants in 41.86% of subjects, with 20.93% carrying pathogenic variants, 9.88% likely pathogenic, and 11.05% variants of uncertain significance (VUS). Frequently identified pathogenic variants were *c.654_656del p.(Gly219del)* in *LDLR* and *c.10580G>A p.(Arg3527Gln)* in *APOB*, which are both linked to the founder effect. Genetic testing led to a reassessment of Dutch Lipid Clinic Network scores, increasing the number of individuals classified as “Definite FH” by 86.2%. Conclusions: The increasing use of NGS in FH has enhanced diagnostic capabilities and suggests population-specific genetic patterns. However, it also increases VUS detection, for which reclassification rates are still low and require strenuous efforts. Moreover, despite the benefits of genetic testing, significant gender disparities remain and require further attention.

## 1. Introduction

Familial hypercholesterolemia (FH) is one of the primary causes of congenital hypercholesterolemia. It is a common autosomal dominant disorder marked by persistently high levels of low-density lipoprotein cholesterol (LDL-C), significantly increasing the risk of atherosclerosis and early coronary heart disease [1,2]. While FH is often caused by known pathogenic or likely pathogenic variants in genes such as the LDL receptor (*LDLR*), apolipoprotein B-100 (*APOB*), or Proprotein convertase subtilisin/kexin type 9 (*PCSK9*), many cases involve variants of uncertain significance (VUS) [3]. VUS present challenges for cardiogenetics due to the lack of sufficient evidence for classification as either pathogenic or benign, complicating genetic counseling and clinical decisions. Although the American College of Medical Genetics and Genomics (ACMG) provides guidelines for variant classification, reclassification rates remain slow, underscoring the need for further research into the potential pathogenicity of VUS [4].

Although genetic testing for FH has become increasingly common, it frequently yields VUS, complicating diagnosis and patient communication. Approximately 40% of patients receive VUS results, highlighting the need for meticulous interpretation [5]. Pre-test counseling is essential to inform patients about the potential for VUS findings adequately. To enhance diagnostic clarity, it is advisable to disclose results only for VUS with a high suspicion of pathogenicity and to conduct segregation analysis in family members to aid in reclassification. As genetic testing technology advances, the effective management of VUS becomes increasingly critical due to the growing prevalence of these variants [4]. Therefore, this study aims to characterize the spectrum of genetic variants associated with FH in a cohort of Lithuanian patients and to assess the potential clinical significance of these variants through in silico analysis and comparison with existing databases.

## 2. Materials and Methods

The study was approved by the Vilnius (Lithuania) regional bioethics committee (permit number 158200-18/5-1010-538, issued 18 May 2018). All patients involved in the study signed an informed consent form.

Retrospective data analysis was performed on data from 172 patients consulted for suspected FH at the Vilnius university hospital Santaros clinics (VUHSC). An initial FH diagnosis was established based on clinical DLCN criteria prior to the initiation of genetic testing. This allowed for a provisional classification into FH likelihood categories based on clinical grounds:

- Definite FH (DLCN score > 8)
- Probable FH (DLCN score 6–8)
- Possible FH (DLCN score 3–5)
- Unlikely FH (DLCN score < 3)

Subsequently, comprehensive genetic testing was conducted for all subjects, and the results were integrated into the diagnostic assessment by updating the DLCN scores where applicable.

All patients in this study were evaluated using the highest documented LDL-C concentration prior to the initiation of any lipid-lowering treatment. For patients with established atherosclerotic cardiovascular disease (ASCVD), this was defined as the LDL-C level recorded either before the cardiovascular event or, if unavailable, during the acute event. This approach is feasible in VUHSC, where lipid profiling (including LDL-C measurement) is routinely performed for all patients presenting with acute coronary syndrome. The study does not include LDL-C values influenced by any ongoing lipid-lowering therapy.

Inclusion criteria:

- Age from 0 to 17 years for children and from 18 to 85 for adults.
- Elevated LDL-C (>4.9 mmol/L in adults and >3.9 mmol/L in children).
- Clinical suspicion of FH.
- Availability of data for initial DLCN scoring in adult patients.

Exclusion criteria:

- Secondary causes of hyperlipidemia (e.g., untreated hypothyroidism, cholestasis, and nephrotic syndrome).
- End-stage oncological or somatic disease.
- Pregnancy.
- Clinically significant cerebrovascular disease.
- Inability to provide informed consent by themselves or their legal guardian.

Next-generation sequencing (NGS) has been chosen as the most appropriate technique for genetic testing. Genomic deoxyribonucleic acid (DNA) was enzymatically fragmented, and regions of interest were enriched using DNA capture probes. The final indexed libraries were sequenced on an Illumina platform. Data analysis was performed using validated in-house software, including alignment to the hg19 human reference genome (Genome Reference Consortium GRCh37), variant calling, and annotation. For the gene panel, the coding regions, 10 bp of flanking intronic sequences, and known pathogenic/likely pathogenic variants (coding and non-coding) of the *ABCA1*, *ABCC2*, *ABCD1*, *ABCD4*, *ADA*, *AGA*, *AGL*, *AGPS*, *ALAD*, *ALAS2*, *ALDOA*, *ALDOB*, *ALPL*, *APOA2*, *APOA5*, *APOB*, *APOC2*, *APOE*, *ARG1*, *ARSA*, *ARSB*, *ASAH1*, *ASL*, *ASS1*, *ATP7A*, *ATP7B*, *BCKDHA*, *BCKDHB*, *CBS*, *CETP*, *CLN3*, *CLN5*, *CLN6*, *CLN8*, *CPOX*, *CPS1*, *CTNS*, *CTSA*, *CTSD*, *CTSK*, *CYP11B1*, *CYP17A1*, *CYP19A1*, *CYP21A2*, *DBT*, *DHCR7*, *ENO3*, *ENPP1*, *EPHX2*, *FECH*, *FGF23*, *FUCA1*, *G6PC*, *G6PD*, *GAA*, *GALC*, *GALE*, *GALK1*, *GALNS*, *GALT*, *GBA*, *GBE1*, *GHR*, *GK*, *GLA*, *GLB1*, *GM2A*, *GNPAT*, *GNPTAB*, *GNPTG*, *GNS*, *GUSB*, *GYG1*, *GYS2*, *HCFC1*, *HEXA*, *HEXB*, *HFE*, *HJV*, *HGD*, *HGSNAT*, *HMBS*, *HPRT1*, *HSD3B2*, *HYAL1*, *IDS*, *IDUA*, *ITIH4*, *KHK*, *LAMP2*, *LCAT*, *LDHA*, *LDLR*, *LIPA*, *LIPI*, *LMBRD1*, *LPA*, *LPL*, *MAN2B1*, *MANBA*, *MCOLN1*, *MFSD8*, *MMACHC*, *MMADHC*, *NAGA*, *NAGLU*, *NAGS*, *NEU1*, *NPC1*, *NPC2*, *OTC*, *PAH*, *PEX1*, *PEX10*, *PEX12*, *PEX13*, *PEX14*, *PEX16*, *PEX19*, *PEX2*, *PEX26*, *PEX3*, *PEX5*, *PEX6*, *PEX7*, *PFKM*, *PGAM2*, *PGK1*, *PGM1*, *PHKA1*, *PHKA2*, *PHKB*, *PHKG2*, *PKLR*, *POR*, *PPOX*, *PPP1R17*, *PPT1*, *PRKAG2*, *PYGL*, *PYGM*, *RBCK1*, *SGSH*, *SLC17A5*, *SLC25A13*, *SLC25A15*, *SLC25A36*, *SLC2A1*, *SLC2A2*, *SLC2A3*, *SLC3A1*, *SLC3A2*, *SLC40A1*, *SLC6A19*, *SLC7A7*, *SLC7A9*, *SLCO1B1*, *SLCO1B3*, *SMPD1*, *SUMF1*, *TFR2*, *TPP1*, *UGT1A1*, *UMPS*, *UROD*, *UROS*, *IVD*, *DLX4*, *ANTXR2*, *ABCB4*, *ABCG5*, *ABCG8*, *ACAT1*, *AGXT*, *ALDH4A1*, *ALG3*, *LDLRAP1*, *BTD*, *CD320*, *CPT1A*, *DDC*, *DIABLO*, *DNAJC5*, *DPYD*, *ETHE1*, *FAH*, *FBP1*, *GAMT*, *GATM*, *GYS1*, *HLCS*, *HPD*, *LIPC*, *MMAA*, *MMAB*, *MMUT*, *PCSK9*, *PDHB*, *PNPO*, *PSAP*, *SI*, *SLC22A5*, *SLC25A20*, *SLC37A4*, *SLC6A8*, and *TAT* genes were targeted for analysis.

Although patients were tested for all 206 genes in the panel, only variants in genes known to be associated with FH—specifically *LDLR*, *APOB*, *PCSK9*, and *LDLRAP1*—were included in the final analysis.

While assessing the prevalence of ASCVD among subjects in FH-related genes, pediatric patients were excluded, as ASCVD is not typically observed or diagnosed in this age group.

All identified variants are evaluated regarding their pathogenicity and causality and categorized into three groups: (1) pathogenic, (2) likely pathogenic, and (3) variants of uncertain significance. Subjects without detected FH-related variants were classified as group (4) No mutation. Orthogonal methods confirmed variants with low quality and/or unclear zygosity. Consequently, a specificity of >99.9% for all reported variants was warranted. The copy number variation (CNV) detection software had a sensitivity above 95% for all homozygous deletions and heterozygous deletions/duplications spanning at least three consecutive exons.

## 3. Statistical Analysis

Statistical data analysis was performed using R (v. 4.0.4). Descriptive statistical methods were used to estimate the variables. Mean, standard deviation (SD), and number of available observations were calculated and reported for the quantitative variables. Categorical variables are expressed as absolute numbers (N) and percentages (%). A one-way ANOVA test was performed for normally distributed quantitative variables to compare means across multiple groups. The non-parametric Mann–Whitney U test was used to compare quantitative variables between two groups. Normality was tested using the Shapiro–Wilk test. A *p*-value less than 0.05 was considered significant.

## 4. Results

The study cohort included 172 patients, 83 (48.26%) men and 89 (51.74%) women. The mean age of the subjects was 36.69 ± 18.33 years. The mean plasma LDL-C concentration of the cohort was 6.75 ± 2.69 mmol/L. The mean LDL-C concentration in males was 6.77 ± 2.82 mmol/L, while in females it was 6.73 ± 2.58 mmol/L (*p* > 0.05).

The mean age at FH diagnosis significantly differed between the sexes: 37 years for men and 46.0 years for women (*p* = 0.033).

**Figure 1:**
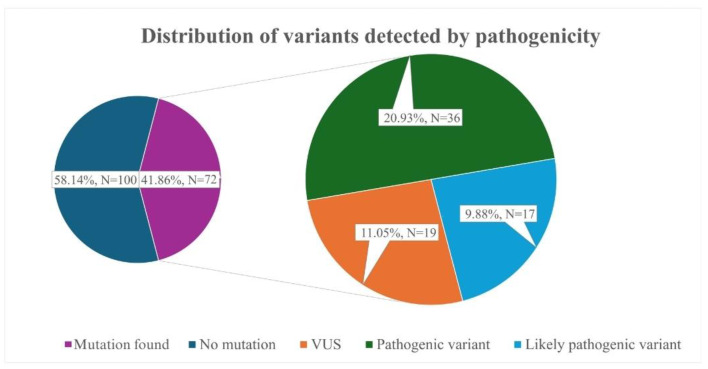
Distribution of variants detected by pathogenicity.

In 41.86% of subjects (N = 72), variants in FH-causing genes were found. Among individuals, 20.93% (N = 36) of individuals had variants classified as pathogenic, 9.88% (N = 17) had likely pathogenic variants, and 11.05% (N = 19) had variants of uncertain significance (VUS) (Figure 1).

The pathogenicity of the mutation variants detected in the subjects was analyzed based on American College of Medical Genetics and Genomics (ACMG) criteria. Thirty-six pathogenic mutation variants were identified: Twenty-eight in the *LDLR*, seven in the *APOB*, and one in the *LDLRAP1*. The most frequently identified pathogenic variants were *c.654_656del p.(Gly219del)* (N = 5), *loss of exons 7-14* (N = 5), and *c.910G>A p.(Asp304Asn)* (N = 5) in the *LDLR* and variant c.10580G>A p.(Arg3527Gln) (N = 7) in the *APOB* (Table 1).

Seventeen variants have been identified as likely pathogenic. All variants were detected in the *LDLR*. The most frequently detected (N = 6) likely pathogenic variant was *c.[941-1_946del]* in *LDLR* (Table 1).

Nineteen patients with VUS in genes associated with FH were identified. Most of the subjects were found to have VUS in the *APOB* gene (N = 14). The most frequent variants found in *APOB* were *c.2630C>T p.(Pro877Leu)* (N = 3) and *c.5066G>A p.(Arg1689His)* (N = 3). Five VUS in *LDLR* were found: *c.949G>A p.(Glu317Lys)* (N = 1), *c.58G>A*, *p.(Gly20Arg)* (N = 1), *c.1210A>C p.(Thr404Pro)* (N = 1), *c.1049G>C p.(Arg350Pro)* (N = 1), and *c.1217G>A p.(Arg406Gln)* (N = 1). No VUS in *LDLRAP1* and *PCSK9* were present in our cohort. In addition, 33.33% (5 out of 15 adult carriers) of patients with identified VUS in FH-related genes were diagnosed with ASCVD (Table 1).

**Figure 2:**
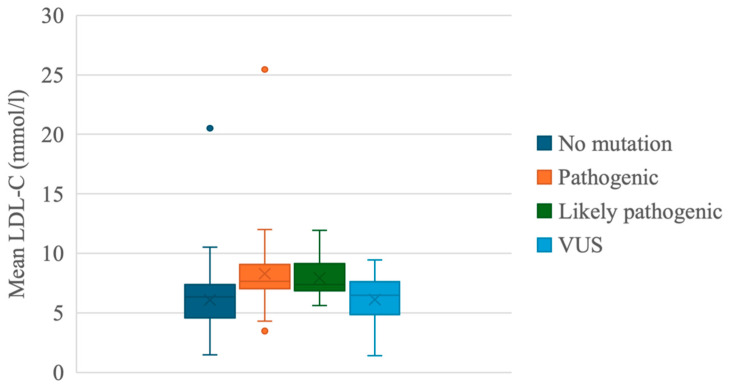
Distribution of mean low-density lipoprotein cholesterol (LDL-C) concentrations by the pathogenicity of the variant detected in all subjects.

The mean LDL-C concentration of subjects with no pathogenic variants was 6.09 ± 2.34, while those with pathogenic or likely pathogenic variants had mean LDL-C concentrations of 8.31 ± 3.39 and 7.95 ± 1.65, respectively. In patients with VUS variants, the mean LDL-C was 6.14 ± 2.23. While carriers of pathogenic and likely pathogenic variants had significantly higher mean LDL-C (respectively, *p* < 0.001 and *p* = 0.027) compared to individuals with no detected variants, there was no significant association (*p* = 0.1) between carriers of VUS and subjects who had no detected variants in FH-related genes (Figure 2). Two notable outliers were observed: one individual with a novel homozygous LDLRAP1 variant (NM_015627.2:c.488A>C; p.Gln163Pro) exhibited an exceptionally elevated LDL-C concentration of 25.45 mmol/L, while another subject without any detected FH-related gene variants had an LDL-C level of 20.52 mmol/L.

**Figure 3:**
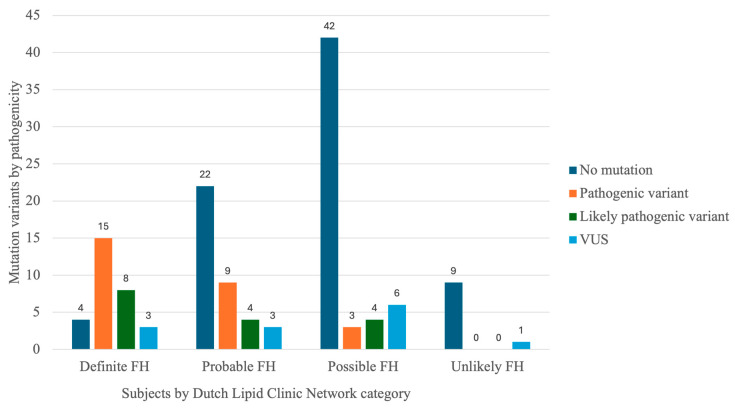
Pathogenicity of variants detected in adult subjects according to the Dutch Lipid Clinic Network Criteria.

The analysis of adult subjects using the Dutch Lipid Clinic Network (DLCN) criteria revealed that the highest prevalence of pathogenic, likely pathogenic, and VUS was observed in the “Definite FH” group, with rates of 48.28% (N = 14), 24.14% (N = 7), and 13.79% (N = 4), respectively. In the “Probable FH” group, pathogenic variants were present in 23.68% (N = 9) of cases, likely pathogenic in 7.89% (N = 3), and VUS in 10.53% (N = 4). Meanwhile, 90% (N = 9) of individuals in the “Unlikely FH” group were found to have no detectable variants in FH-related genes. Pediatric subjects were excluded as DLCN criteria do not apply to them (Figure 3).

Before genetic testing, 29 individuals were classified as belonging to the “Definite FH” category. Following genetic testing, DLCN scores were reevaluated, increasing the number of individuals in the “Definite FH” group to 54, leading to an 86.2% rise in the total of the “Definite FH” category.

## 5. Discussion

FH is an autosomal dominantly inherited genetic disorder, the primary manifestation of which is an increase in LDL-C levels [6]. Most commonly, FH is caused by mutations in the genes encoding *LDLR*, *ApoB*, and *PCSK9*, factors involved in LDL-C metabolism [7,8]. Variants in the *LDLR*, *APOB*, and *PCSK9* genes have the typical consequence of impaired LDL-C clearance. Therefore, LDL-C and total cholesterol increase is observed in patients with FH [3]. The recessive form of the disease, caused by a mutation in the *LDLRAP1* gene, is much less frequent and is more prevalent in regions with high rates of consanguineous marriages [3,9].

A total of 36 pathogenic variants were detected in this study (Table 1). Of these, 27 were detected in *LDLR*, 8 in *APOB*, and 1 in *LDLRAP1* genes. One frequently identified (N = 5) variant in *LDLR* was *NM_001195798: c.651_653del*, *NP_000518.1: p.(Gly219del)*, *rs121908027*. Evidence suggests this mutation may be associated with the founder effect in Ashkenazi Jews of Lithuanian origin [10]. Another prominent variant involved the deletion of exons 7–14 in the *LDLR* (N = 5). The literature describes that mutations with such a large genetic rearrangement may account for about 5% of all FH cases [11]. In our cohort, the prevalence of exon 7–14 deletion in the *LDLR* gene accounted for 0.069% (5 out of 72) of the total variants detected. However, in the study by Nissen and colleagues, large-scale exon deletion mutations accounted for 3.1% of mutations in Danish populations, which contrasts with the results obtained in similar studies in Canada and Norway. According to the authors, such differences highlight the need for genetic studies in different populations to elucidate the genetic spectrum of FH [11]. This variant has been documented in the literature as removing the epidermal growth factor (EGF) precursor homology domain, which is essential for LDL receptor function [12,13]. This leads to significantly elevated LDL-C levels, a poor response to statin therapy, and early-onset atherosclerosis, as illustrated in a case reported by Agirbasli et al. involving a homozygous FH patient with extensive xanthomas and markedly high LDL-C levels [13]. The most frequent variant detected overall was *NM_000384.3:c.10580G>A*, *NP_000375.3:p.(Arg3527Gln)* in the *APOB* gene (N = 7). Some authors suggest that this variant is related to the founder effect and is specific to the Amish community in Lancaster, Pennsylvania. In this community, the prevalence of this mutation exceeds the prevalence of FH in the general population and may be up to 12% [14].

A notable outlier was identified within the pathogenic variants, where one subject exhibited an exceptionally elevated LDL-C concentration of 25.45 mmol/L. This individual was found to carry a novel pathogenic variant in the *LDLRAP1* gene. Upon further investigation, a homozygous variant *NM_015627.2: c.488A>C*, *NP_056442.2: p.(Gln163Pro)* was detected, consistent with a diagnosis of autosomal recessive hypercholesterolemia (ARH). This variant was first reported in the literature by Z. Petrulioniene and colleagues [15]. Another outlier is an individual with no detected variant in FH-related genes, where LDL-C concentration was 20.52 mmol/L. According to the DLCN criteria this case would be classified as definite FH. However, the high LDL-Ch could likely be due to the combined effects of various environmental factors traditionally associated with hypercholesterolemia, such as obesity, harmful habits, etc. [16,17]. In contrast, an individual with an identified pathogenic variant had a relatively low LDL-C level, deviating from the group average, likely since this subject was of pediatric age. Our study identified 17 likely pathogenic variants, all in the *LDLR* (Table 1). Variant *c.[941-1_946del]* in the *LDLR*, which is not described in the scientific literature, was detected six times.

Although genetic testing is a reliable approach for diagnosing the disease, it may fail to identify primary mutations in 20–40% of FH cases [18]. One of the several limitations associated with genetic testing is reduced penetrance, which means that not all individuals with FH-associated genetic variants exhibit high cholesterol levels or apparent clinical symptoms. Additionally, lipid-lowering therapy (LLT) can mask hypercholesterolemia and coronary heart disease phenotypes, further complicating detection. The reliance on family history presents additional challenges, as self-reported information may be inaccurate or unavailable, and many children with molecularly confirmed FH lack a documented family history of cardiovascular disease [19].

Diagnosing FH in children is particularly difficult, as traditional criteria such as the DLCNC are not valid in pediatric cases, necessitating reliance on family history and serial LDL-C measurements, which may be inconsistent [19,20]. Furthermore, variability in LDL-C levels among individuals with FH mutations can hinder identification, as a significant proportion have LDL-C levels below commonly used diagnostic cutoffs [19]. Overlap in LDL-C levels between individuals with and without FH pathogenic variants further complicates diagnosis, particularly in adults, as LDL-C levels tend to rise with age [19,21]. A study by Huigen et al. found that in their cohort, 15% of the untreated patients with genetically confirmed FH did not have severely increased LDL-C levels [21]. While genetic testing can help distinguish individuals with FH from those with elevated cholesterol due to other causes, it remains an imperfect tool. The lack of international consensus on the best diagnostic criteria for FH, combined with the reliance on physical features, premature coronary artery disease (CAD), and family history, reduces diagnostic sensitivity. As a result, many individuals with FH remain undiagnosed. While genetic testing is crucial in identifying individuals who might otherwise remain undiagnosed, an integrated approach that combines genetic, clinical, and biochemical assessments is necessary to improve FH detection and management [19].

Over the last few years, next-generation sequencing (NGS) has become increasingly crucial for diagnosing and investigating FH. It allows targeted analysis of a specific set of genes, the entire coding regions (whole exome), or the whole genome sequence, leading to increased diagnoses [22,23]. Nonetheless, it has also raised the frequency of identifying VUS, which are often novel and lack sufficient evidence for accurate classification, posing a growing diagnostic challenge [23].

In our study cohort, 26.39% (19 out of 72) of subjects presenting with clinical FH phenotype after genetic testing were found to have VUS in genes associated with FH. VUS and pathogenic and likely pathogenic variants were identified in the *LDLR*, *APOB* and *LDLRAP1* genes, yet no variants in the *PCSK9* gene were found. All the VUS found were missense single nucleotide substitutions. Most VUS were present in *APOB* (N = 14). The most frequently recurring VUS was *APOB c.2630C>T p.(Pro877Leu)*, which has been reported twice in the ClinVar database, followed by *APOB c.5066G>A p.(Arg1689His)*, which has been documented three times. Despite currently being classified as VUS, both variants in our study cohort were associated with the development of ASCVD as well as other VUS, whose incidence in our study cohort was less frequent: *LDLR* c.58G>A, p.(Gly20Arg) and *APOB c.7724A>T p.(Lys2575Ile)* (Table 1). 21.05% (4 out of 19) of the VUS detected (Table 1) were found in pediatric patients: *c.1210A>C p.(Thr404Pro)* (N = 1) and *c.949G>A p.(Glu317Lys)* (N = 1) in *LDLR* and *c.2450T>C p.(Ile817Thr)* (N = 2) in the *APOB* gene. While none of the children were found to have ASCVD at the time of the diagnosis, all had elevated LDL-C levels for their age. Therefore, tracking the clinical manifestation of their symptoms could be beneficial during a longitudinal study. Emerging evidence from VUS research indicates a potential association between VUS in the *LDLR* gene and adverse cardiovascular outcomes in FH patients. These findings suggest that incorporating VUS analysis into genetic testing can enhance diagnostic accuracy and risk stratification, improving clinical management of FH [24].

When a VUS is identified, it poses challenges for cascade testing, as the implications of such variants remain unclear [25]. This uncertainty particularly extends to prescribing statins to children, where the balance between potential long-term benefits and risks is not yet fully established [26]. Furthermore, elevated levels of lipoprotein(a) (Lp(a)) have also been identified as independent predictors of cardiovascular disease in FH patients, regardless of the specific FH causative variant present [27]. Despite this, some researchers argue that incorporating VUS in FH cascade screening might benefit further variant reclassification [28].

Nonetheless, the majority of VUS in our cohort were detected in *APOB*. Data from the ClinVar database indicate that 58% of reported *APOB* variants linked to FH are classified as VUS, compared to only 8% of *LDLR* variants. Furthermore, some missense *APOB* variants are associated with conditions such as hypocholesterolemia or hypobetalipoproteinemia, while others display incomplete penetrance. This underscores the need for functional characterization of *APOB* variants to better delineate their pathogenic potential [29]. However, *APOB* and *PCSK9* genes, due to their high polymorphism and less impact on LDLR activity, are more likely to yield VUS results than those in the *LDLR* gene, which are also more frequently reclassified [30].

However, VUS carriers in our cohort did not show significantly elevated mean LDL-C levels compared to individuals without detected variants in FH-related genes (Figure 2). When analyzing individuals’ clinical FH expression based on their DLNC score, several VUS carriers fell into the “Definite” and “Probable” FH categories. Though this classification does not confirm variant pathogenicity, it raises the question of whether carriers of VUS in these categories should be further analyzed individually.

In addition to the scientific and clinical implications of analyzing VUS, the psychological value of such findings for patients and their families should be considered. The uncertainty surrounding a VUS diagnosis often leads to considerable emotional distress. As demonstrated in a study by Tsai et al., reclassifying VUS plays a crucial role in alleviating this uncertainty, thereby improving patients’ ability to cope with their condition and enhancing overall psychological well-being [31].

Nonetheless, genetic testing is crucial for a definite FH diagnosis. Using only the Dutch Lipid Clinic Network criteria for diagnosis, 30 subjects were included in the “Definite HH” group, whereas after genetic testing, an additional 25 subjects were included in this group. Similar results were observed in a study published by Diboun and colleagues in 2022 [1].

However, in our study, women received an FH diagnosis significantly later than men. Similar tendencies have already been shared in current literature, stating that females received diagnosis and, consequently, treatment three to seven years later than men. This further emphasizes the need for extensive studies of the FH genetic profile [32].

## 6. Conclusions

The growing application of NGS in FH has expanded diagnostic capabilities while providing more insight into the genetic spectrum of the disease. The substantial prevalence of variants associated with the founder effect in our Lithuanian cohort further emphasizes the need for population-specific studies. However, it also increased the detection of VUS, presenting a challenge for accurate classification, thus underscoring the need for comprehensive functional characterization of VUS to improve diagnostic accuracy, risk stratification, and clinical management. Although genetic testing increases confirmed FH cases substantially, there are still significant gender disparities that need to be addressed. Thus, further larger-scale studies are necessary to enhance our understanding of the FH genetic spectrum.

## 7. Limitations of the Study

The present study has several limitations that should be acknowledged. Firstly, this study employed a retrospective design and lacked long-term follow-up data to assess the trajectory of clinical outcomes and validate the pathogenicity of identified variants. Therefore, the ability to establish causal relationships between genetic findings and clinical phenotypes is restricted. Secondly, our study cohort was derived from a single tertiary care center and may not fully represent the broader Lithuanian population, potentially affecting the generalizability of the findings. Hence, further research focusing on longitudinal data and nationwide screening systems is pivotal in providing better insight into the genetic FH variability. Incorporating a healthy control group in this study presents challenges as the availability of costly genetic testing is limited.

**Key points:**

- Our study identified 36 pathogenic variants in the *LDLR*, *APOB,* and *LDLRAP1* genes. Frequently identified variants were *LDLR c.654_656del p.(Gly219del)* and *APOB c.10580G>A p.(Arg3527Gln)*, which are both associated with the founder’s effect.
- Genetic testing increased the number of patients classified as “FH” according to the Dutch Lipid Clinic Network criteria by nearly 86.2% in our cohort.
- Women in our cohort were diagnosed with FH nine years later than men, supporting claims of gender disparities in FH diagnostics.

## Figures and Tables

**Table 1:** Prevalence of variants identified in causing FH-related genes in the Lithuanian cohort.

Gene	Variant	Genomic/Variant Coordinates	Pathogenicity (ACMG *)	Number of Carriers	Incidence of ASCVD **
*LDLR ^^^*	loss of exons 7–14	chr19:11221280-11231311	P *^^^^*	5 ^#^ (3) ^##^	0
*LDLR*	*c.301G>A p.(Glu101Lys)*	NM_000527.2:c.301G>A	P	1	0
*LDLR*	*c.654_656del p.(Gly219del)*	NM_000527.2:c.654_656del	P	5 (1)	0
*LDLR*	*c.910G>A p.(Asp304Asn)*	NM_000527.2:c.910G>A	P	5 (2)	0
*LDLR*	*c.1187-10G>A*	NM_000527.2:c.1187-10G>A	P	1	0
*LDLR*	*c.[1106del];[1106=] p.[(Val369GlyfsTer44)];[(Val369=)]*	NM_000527.5:c.[1106del];[1106=]	P	3	0
*LDLR*	*c.[244T>C];[244=] p.[(Cys82Arg)];[(Cys82=)]*	NM_000527.5:c.[244T>C];[244=]	P	1	0
*LDLR*	*c.[1013G>A];[1013=] p.[(Cys338Tyr)];[(Cys338=)]*	NM_000527.5:c.[1013G>A];[1013=]	P	1	0
*LDLR*	*c.[1775G>A];[1775=] p.[(Gly592Glu)];[(Gly592=)]*	NM_000527.5:c.[1775G>A];[1775=]	P	3	1
*LDLR*	*c.[986G>A];[986=] p.[(Cys329Tyr)];[(Cys329=)]*	NM_000527.5:c.[986G>A];[986=]	P	1	0
*LDLR*	*c.651_653del p.(Gly219del)*	NM_001195798: c.651_653del	P	1	1
*LDLR*	*c.1187-10G>A*	NM_000527.2:c.1187-10G>A	P	1	0
*APOB ^§^*	*c.10580G>A p.(Arg3527Gln)*	NM_000384.2:c.10580G>A	P	7 (2)	2
*LDLRAP1 ^§§^*	*c.488A>C (p.(Gln163Pro))*	NM_015627.2:c.488A>C	P	1 (1)	0
*LDLR*	*c.941-1_946del*	NM_000527.2:c.941-1_946del	LP	6 (2)	0
*LDLR*	c.80dup p.(Cys27Trpfs*25)	NM_000527.2:c.80dup	LP	1	0
*LDLR*	*c.986G>A p.(Cys329Tyr)*	NM_000527.2:c.986G>A	LP	3	0
*LDLR*	*c.1222G>A p.(Glu408Lys)*	NM_000527.2:c.1222G>A	LP	1	0
*LDLR*	*c.418G>A p.(Glu140Lys)*	NM_000527.2:c.418G>A	LP	1	0
*LDLR*	c.1303del p.(Glu435Argfs*16)	NM_000527.2:c.1303del	LP	1	0
*LDLR*	*c.1013G>A p.(Cys338Tyr)*	NM_000527.2:c.1013G>A	LP	1	1
*LDLR*	*c. 1027 G>A p.(Gly343Ser)*	NM_000527.2:c. 1027 G>A	LP	1 (1)	0
*LDLR*	c.1183del p.(Val395Trpfs*18)	NM_000527.2:c.1183del	LP	2 (1)	0
*LDLR*	*c.1217G>A p.(Arg406Gln)*	NM_000527.2:c.1217G>A	VUS ^†^	1	0
*LDLR*	*c.1049G>C p.(Arg350Pro)*	NM_000527.2:c.1049G>C	VUS	1	1
*LDLR*	*c.1210A>C p.(Thr404Pro)*	NM_000527.5:c.1210A>C	VUS	1 (1)	0
*LDLR*	*c.58G>A p.(Gly20Arg)*	NM_000527.2:c.58G>A	VUS	1	1
*LDLR*	*c.949G>A p.(Glu317Lys)*	NM_000527.5:c.949G>A (p.Glu317Lys)	VUS	1 (1)	0
*APOB*	*c.4027C>T p.(Pro1343Ser)*	NM_000384.2:c.4027C>T	VUS	1	0
*APOB*	*c.7615G>A p.(Val2539Ile)*	NM_000384.2:c.7615G>A	VUS	1	0
*APOB*	*c.2630C>T p.(Pro877Leu)*	NM_000384.2:c.2630C>T	VUS	3	1
*APOB*	*c.7724A>T p.(Lys2575Ile)*	NM_000384.2:c.7724A>T	VUS	1	1
*APOB*	*c.5066G>A p.(Arg1689His)*	NM_000384.2:c.5066G>A	VUS	3	1
*APOB*	*c.13480_13482del p.(Gln4494del)*	NM_000384.2:c.13480_13482del	VUS	1	0
*APOB*	*c.2248A>G p.(Met750Val)*	NM_000384.2:c.2248A>G	VUS	1	0
*APOB*	*c.2450T>C p.(Ile817Thr)*	NM_000384.2:*c.2450T>C*	VUS	2 (2)	0
*APOB*	*c.8747C>A p.(Ala2916Asp)*	NM_000384.2:c.8747C>A	VUS	1	0

* ACMG, American College of Medical Genetics and Genomics; ** ASCVD, atherosclerotic cardiovascular disease; *^^^ LDLR*, low-density lipoprotein receptor-encoding gene; *^^^^* P, pathogenic variant; ^#^ x, number of adult carriers; ^##^ (x), number of child carriers; *^§^ APOB*, apolipoprotein B-encoding gene; *^§§^ LDLRAP1*, low-density lipoprotein receptor adapter protein 1; ^†^ VUS, variant of uncertain significance.

## Data Availability

Data is contained within the article.
